# One Health in the public mind: Narrative constructions of zoonotic spillovers

**DOI:** 10.1016/j.onehlt.2026.101411

**Published:** 2026-04-14

**Authors:** Elizabeth A. Shanahan, Xin Han, Sarah Salam, Savanna Washburn, Sally K. Slipher

**Affiliations:** a Department of Political Science, Montana State University, Bozeman, MT, USA; b Social Data Collection and Analysis Services, Montana State University, Bozeman, MT, USA

**Keywords:** One Health, Zoonotic spillover, Narrative Policy Framework, Health policy, Problem definition, Human–animal–environment interaction

## Abstract

The COVID-19 pandemic heightened global attention to the One Health (OH) framework—a multisectoral, multidisciplinary approach that integrates human, animal, and environmental health to address challenges such as zoonotic spillovers. While OH has gained traction in scientific and policy arenas, little is known about how the public understands OH issues. Such problem definitions matter because they are theorized to align with logical, congruent solutions. This study examines (1) how often people identify OH dimensions (human, animal, environment) when defining zoonotic spillovers; (2) whether narrative content varies between whole and partial OH definitions; and (3) whether narrative content is congruent with their problem definitions. Based on a survey from residents of eastern coastal Australia (*n* = 1549, after data cleaning), we coded open-ended responses about problem definitions, heroes, and solutions, and analyzed results with Poisson generalized linear modeling of counts. A small portion of respondents (14%) articulated a full OH problem definition; the majority (75%) emphasized human dimensions, while just 11% highlighted environmental or animal aspects. Respondents using a OH definition were more likely to identify diverse heroes spanning human, environmental, and animal organizations, and to propose environmental solutions compared with those using human-centered definitions. Evidence of congruence between problem definitions and solutions emerged, with OH problem definitions associated with a multisectoral array of heroes. Overall, public conceptions of zoonotic spillovers remain fragmented, reflecting limited uptake of integrative OH perspectives. Strengthening public understanding of OH and fostering coherent, systems-based narratives are essential for enhancing policy legitimacy and advancing preventive, cross-sectoral approaches to OH issues more broadly.

## Highlights

• Despite global adoption of a One Health strategy, public use of One Health dimensions to describe zoonotic spillovers remains low.
• Public narratives on zoonotic spillover solutions and responsible actors remain fragmented and siloed.
• Those with One Health understanding use diverse heroesand more environmental solutions, keys to multisectoral prevention.

## 1 Introduction

The global experience of COVID-19 has accelerated the recognition among governments, international organizations, and scientific communities of the need for a One Health (OH) framework in addressing emerging infectious diseases (EIDs) [1]. OH is fundamentally a systems-based approach that recognizes the interdependence of human, animal, and environmental health, thereby requiring integrated, multisectoral, and interdisciplinary responses to global health challenges [2]. Recent years have seen a proliferation of national and regional OH strategic action plans [3], yet implementation remains a challenge. Scholars have noted the persistent structural and institutional silos across public health, animal welfare, and environmental governance that limit collaboration and resource-sharing, as well as lack of political will and financial resources [4]. These divisions are not only bureaucratic but also epistemological, as disciplinary training from molecular biology to sociology produces distinct knowledge cultures that can result in fragmented solutions [5]. While efforts to build systems thinking across sectors and disciplines are focused on coordination, communication, collaboration, and capacity-building [1], public awareness of OH principles remains comparatively low [6], [7].

The extent to which people understand the integrated and interdependent nature of OH issues such as zoonotic spillovers is consequential, because how people think about or define a problem delineates what types of solutions are seen as legitimate to solve the problem [8]. For example, if the problem with zoonotic spillovers is ‘sick animals,’ then logical solutions may include veterinary vaccine campaigns or animal culling. In contrast, if the problem with zoonotic spillovers is attributed to loss of biodiversity, then logical solutions may include land conservation or habitat restoration. The ways in which the public defines problems thus carry significant implications for which OH policy solutions are politically and socially viable.

In this study, we investigate the extent to which the public conceptualizes zoonotic spillovers in a OH framework. Specifically, we explore the degree to which participants articulate siloed understandings (focusing solely on one domain—animals, humans, or the environment), partially connected assessments (e.g., animals and humans; animals and environment) or fully integrated OH definition (linking all three domains of animal, human, and environment). In addition to identifying the variation in how people define zoonotic spillovers, we also sought to capture the narratives associated with these problem definitions. The Narrative Policy Framework (NPF) argues that as *homo narrans*, individuals process and communicate policy issues in narrative form [9], [10]. We thus examine how people tell the story of zoonotic spillovers: not only why it is a problem (causal story or problem definition), but also what the solution is (moral) and who should fix it (hero). We analyze how narrative constructions vary by how people define the problem of zoonotic spillovers. In doing so, our study contributes to understanding how people's narratives reflect siloed thinking or an integrated approach, necessary for effective One Health governance.

### 1.1 What is One Health?

One Health is not a new concept; it has just simply been named recently. Indeed, human-animal-environment interconnectedness has been practiced and held as a core value by indigenous peoples across the globe for centuries [11]. Over the last 150 years in the western hemisphere, discoveries at the intersection of veterinary and human medicine advanced the concept of animal-human linkages [12], giving birth to disciplines of immunology, pathology, microbiology, epidemiology, and virology. In 2004, the “One World, One Health” symposium formally expanded the framework to incorporate environmental health, further subdivided into biotic (e.g., ecosystems, biodiversity) and abiotic (e.g., water, climate, soil) dimensions [13], [14]. In the wake of the COVID-19 worldwide pandemic, a quadripartite of global organizations–the FAO, UNEP, WHO, and WOAH– solidified the place of ecosystems in the health system, defining OH as “an integrated, unifying approach that aims to sustainably balance and optimize the health of humans, animals, plants and ecosystems. It recognizes the health of humans, domestic and wild animals, plants and the wider environment (including ecosystems) are closely linked and interdependent” [1], [4]. The OH approach is now used beyond animal-human infectious disease issues to encompass global challenges such as climate change, food security and safety, and antimicrobial resistance [1], [11].

**Fig. 1:**
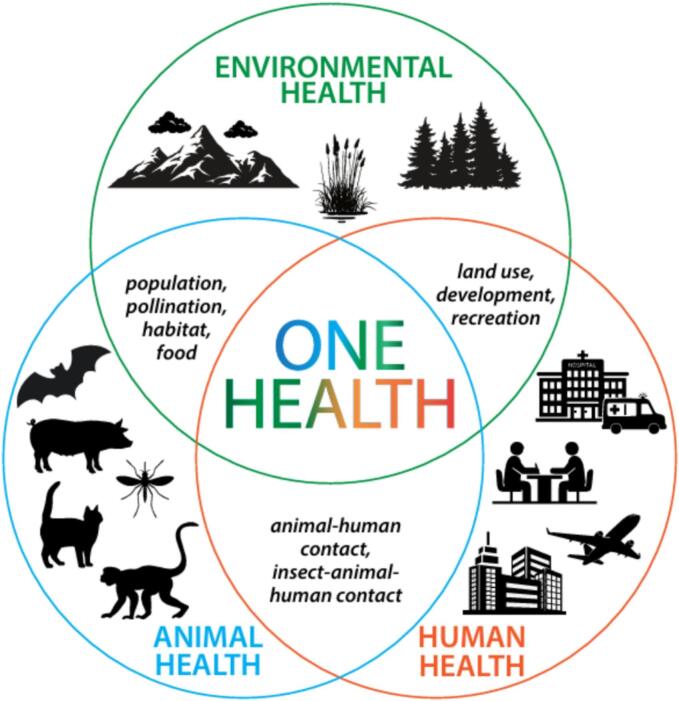
One Health as an interconnected system.

OH is typically rendered as a triple Venn diagram to represent the interconnected nature of the three domains: humans, animals, and the environment (Fig. 1). However, the scholarly works claiming to be OH research remain heavily concentrated in biomedical, veterinary, and disease ecology [15]. This reflects a persistent siloing of research that risks neglecting the social, cultural, and political drivers of zoonotic spillovers [16] and environmental factors [15]. Yet, tackling “wicked problems”—such as pandemics, climate change, and food insecurity—requires a holistic, multisystem approach that integrates not only biological and ecological perspectives but also social and behavioral insights [17].

Among scholars and practitioners already working within OH or adjacent fields there is a growing recognition of the need for greater epistemic diversity [18], [19]. The importance of true integration of varied forms of expertise, ontologies, and epistemologies aim to move beyond technocratic solutions [20], [21]. Such synthesis of perspectives is essential to realize OH's potential as both a scientific paradigm and a governance framework, capable of addressing the multifaceted drivers of health crises in an interconnected world.

### 1.2 Public understanding of One Health

Wildlife are found to be reservoir hosts for many EIDs, and human activities such as land-use change increases human-wildlife contact thereby amplifying the risks of spillover events [22]. And yet, public understanding of these critical linkages remains limited. For instance, despite the proliferation of OH strategic plans following the COVID-19 pandemic, a recent study in the United Kingdom found that lay engagement with OH concepts occurred mainly among individuals who held an a priori interest in nature and animals [23]. Thus, broader awareness of the integrative OH framework is still nascent.

Perceptions from professionals working in OH-related sectors reinforce this conclusion. A multi-country survey of experts and leaders working in animal, human, and environmental health areas in governments, academia, and the private sector found that while most (57.9%, *n* = 99) were familiar with the term “One Health,” relatively few (10.7%, *n* = 16) could fully define or operationalize it [6]. Respondents frequently cited “communication and organizational problems” as barriers to intersectoral collaboration, reflecting the persistence of siloed practices even among OH advocates.

This knowledge gap matters because public comprehension of the interdependence between humans, animals, and the environment is critical for policy support—both normatively and financially. Without public recognition of OH linkages, prevention and mitigation measures may lack support or sustained investment. Thus, improving public understanding is not simply an educational task but a prerequisite for building political will and financial resources for cross-sectoral OH interventions.

### 1.3 Zoonotic spillover as a One Health issue

It is no longer a question of *if* but rather *when* the world will face the next pandemic. As natural habitats undergo continued loss and fragmentation, the boundaries of the wildland–urban interface (WUI) have blurred. Currently, the WUI is home to an estimated 3.5 billion people, representing roughly 44% of the global population [24]. This vast overlap of human and wildlife habitats substantially increases opportunities for zoonotic transmission [25].

More than half of all human infectious diseases originate from non-human animals, a process referred to as zoonotic spillover [26]. Historical outbreaks of Zika, Ebola, and most recently COVID-19 underscore the catastrophic health, economic, and social consequences of such events. While OH is not a discipline per se, it provides a holistic perspective through which to study and address these risks [27]. Importantly, the incorporation of social behavioral sciences in OH scholarship has expanded our predictive capabilities of human responses to spillover events in tandem with attitudes toward prevention and management of zoonotic threats [16].

The Quadripartite's *One Health Joint Plan of Action* [1] and several national level OH strategic plans [3] prioritize prevention of zoonotic spillover as a cornerstone of pandemic preparedness. However, successful implementation depends on understanding how the public, communities, and decision-makers perceive these risks and respond to proposed interventions. Accordingly, research on public understanding of and response to OH is critical for operationalizing and implementing OH into actionable policies. This study contributes to that agenda by examining how people define zoonotic spillovers and tell their story of who should fix the problem and how—an essential step in aligning public discourse with global OH goals.

### 1.4 Problem definitions, the Narrative Policy Framework, and One Health

Problem definitions reflect how individuals and groups make sense of complex phenomena. They are fundamental to the policy process because they shape which solutions are considered legitimate or feasible during political debate [8], [28], [29]. Problem definitions are constructed through multiple sources—interest groups, policymakers, the media, and scientific communities. Yet, how the public defines problems also matters, since public definitions can influence both policy support and compliance.

The Narrative Policy Framework (NPF) [10] provides theoretical scaffolding to analyze how policy problems are communicated and contested. While the NPF has considered connections to framing theory and policy narratives [30], the intersection between problem definitions and causal stories remains undertheorized. Both are social constructions of policy reality: problem definitions specify causes and consequences, while narratives embed these within stories that feature characters and moral lessons. Together, they provide a cognitive and rhetorical structure through which people interpret complex issues. In this sense, villains and victims constitute the *causal story*, while heroes and solutions represent the preferred path forward. A generic narrative template illustrates this structure:

*“[policy issue]* is happening because of *[villain]*, and *[victim]* is suffering as a result. *[hero]* must *[moral/solution].”*

Example 1

*The COVID pandemic [policy issue]* is happening because of *the sale of virus carrying animals in wet markets [villain],* and *people got sick [victim]* as a result. *The government [hero]* must *regulate animal trade [solution]*.

Example 2

*The COVID pandemic [policy issue]* is happening because of *exchanges of infected airborne particles at gatherings such as religious, school, and social events [villain],* and *people got sick [victim]* as a result. *All of us [hero]* must *stay home and wear masks [solution]*.

We posit that OH is a problem definition for global health issues such as EIDs and antimicrobial resistance. A complete OH problem definition would incorporate humans, animals, and the environment simultaneously. By contrast, partial OH definitions would highlight only a subset of these domains (e.g., humans and animals without environmental drivers). In this study, we examine how the narrative components of hero (the actor tasked with solving the problem) and solution (the moral of the story) intersect with problem definitions of zoonotic spillover. This approach allows us to analyze not only *what* the public perceives to be the problem, but also *how* they narratively construct responsibility and prescribe action—insights that are essential for designing effective, publicly legitimate One Health policies.

## 2 Research questions

The purpose of this study is to contribute to the growing literature on the public's understanding of OH. Comparatively little is known within the OH scholarship about how the public conceptualizes OH problems or constructs narratives to explain them. We adopt a novel approach by linking the concept of problem definition with the Narrative Policy Framework [10] in order to capture a robust understanding of how individuals think about the OH issue of zoonotic spillovers. As such, we ask the following research questions:

1. To what extent do individuals use all, some, or none of the OH dimensions (human, animal, environment) when defining the problem of zoonotic spillovers?
2. Does narrative content (i.e., heroes and solutions) vary when comparing a complete OH problem definition to other partial OH problem definitions?
3. To what degree is the narrative content congruent with the accompanying problem definition?

## 3 Methods, data, and analysis

### 3.1 Data collection and survey design

In 2023, we conducted a large-scale survey of 3974 Australian residents aged 18 and older, focusing on three states: Queensland (Qld; *n* = 1093; 28%), New South Wales (NSW; *n* = 1512; 38%), and Victoria (*n* = 1369; 34%). We imposed soft quotas within each state to ensure balance across gender and age categories, thereby enhancing representativeness. Recruitment was conducted by *i*-Link Research Services, an Australian survey firm.

The geographic focus was purposive. The three states contain coastal habitat for *Pteropodid* bats, known to carry a variety of viruses (e.g., Hendra virus [HeV], Australian Bat Lyssavirus [ABLV]). Additionally, Qld and NSW have a long history of HeV (*henipavirus hendraense*) spillovers, and there is concern in Victoria for bat-borne viruses to continue its march south into their state. As such, these three states are excellent case studies and salient contexts for public experience with and perceptions about zoonotic disease.

The survey instrument was designed to elicit qualitative, open-ended responses to capture both problem definitions and narrative constructions. Three key prompts were employed:

Problem Definition: *Viruses are spilling over from animals to humans more frequently than before. Briefly explain why you think this is happening.*

Hero: *Who do you trust the most to best manage the problem of zoonotic viruses?*

Solution: *What do you think ought to be done to prevent the spillover of viruses from animals to humans?*

Data collection procedures comply with the World Medical Association Declaration of Helsinki and was approved by Montana State University Institutional Review Board (ES050521-EX). Informed consent was obtained for all participants, with results being analyzed and discussed only in the aggregate and privacy rights are ensured through the assignment of unique identifiers.

### 3.2 Coding process

We employed both deductive and inductive coding strategies [31] to develop three codebooks for Problem Definitions, Heroes, and Solutions (Supplement A). For Problem Definition, responses were coded deductively into categories that reflect the OH dimensions—human health, animal health (wild or domestic), and environmental health (biotic or abiotic). Responses not fitting these categories were coded as “Other”. While the NPF informed the definitions of hero and solution as constructs, the specific types of heroes and solutions were inductively coded. Through iterative coding and refinement, coders systematically reviewed responses to identify meaningful classifications of Heroes (e.g., government; scientists; human, animal, or environmental organizations) and Solutions (e.g., regulatory, technical/science-based).

Coding was conducted using Qualtrics to facilitate organization and traceability for intercoder reliability analysis. All coders participated in training sessions to standardize definitions of coding categories. To ensure reliability, iterative coding sessions were conducted, starting with randomly selected subsamples (a subset of responses each for problem definition, heroes and solutions) to establish initial codebooks. These codebooks were then refined as additional themes emerged. Independent double-coding of responses was performed, and intercoder reliability was assessed using Cohen's Kappa, which indicated generally strong agreement (e.g., > 0.6) across problem definition, hero, and solution categories (Supplement B).

### 3.3 Data and analysis

We analyzed responses as combinations of Problem Definitions, Heroes, and Solutions. Each response was coded with one Problem Definition, but respondents could mention multiple Heroes or Solutions. Therefore, a single response could yield multiple combinations of narrative components. Responses that did not contain all three components were considered incomplete and dropped from analysis. Responses “I don't know” and nonsensical entries (e.g., a string of letters) were excluded to ensure the dataset accurately reflected informed perspectives. This step was crucial in refining the dataset, enhancing the quality of the analysis, and ensuring that our findings were based on substantive responses rather than uncertainty or lack of knowledge. After data cleaning, a total of 3551 combinations across 1549 participants were used to test for significant associations between categories of Problems, Heroes, and Solutions. There were 255 unique combinations that made up the 3-way contingency table of Problem, Hero, and Solution counts (see Supplement C for examples of observations by subject and unique observations). Data were binary coded (1 = present; 0 = absent) for each category and tallied into 3-way counts to enable statistical modeling (see Supplement D for demographics by Problem Definition).

The cell counts of these combinations of Problem Definition, Hero, and Solution categories were fit with a log-linear model (a generalized linear model on the Poisson link scale) to test if certain combinations of responses appeared together more frequently (Supplement E). A likelihood ratio test was used to test for all three two-way interactions (Problem × Hero; Problem × Solution; Hero × Solution). Although a three-way interaction may also exist, the data were too sparse, thereby preventing robust estimation. Problem Definition group size was used in the model to offset the imbalance of groups, accounting for the fact that uneven numbers of people responded in accordance with different problem definitions (Supplement F).

To further probe associations, we conducted pairwise comparisons of estimated odds ratios, focusing on two sets of contrasts: One Health problem definitions compared to other partial definitions within (a) Hero categories and (b) Solution categories. *P*-values were adjusted for multiple comparisons using the False Discovery Rate method to reduce the risk of Type I error.

## 4 Results

We present our results in order of our three research questions.

### 4.1 To what extent do individuals use all, some, or none of the One Health dimensions when defining the problem of zoonotic spillovers?

**Fig. 2:**
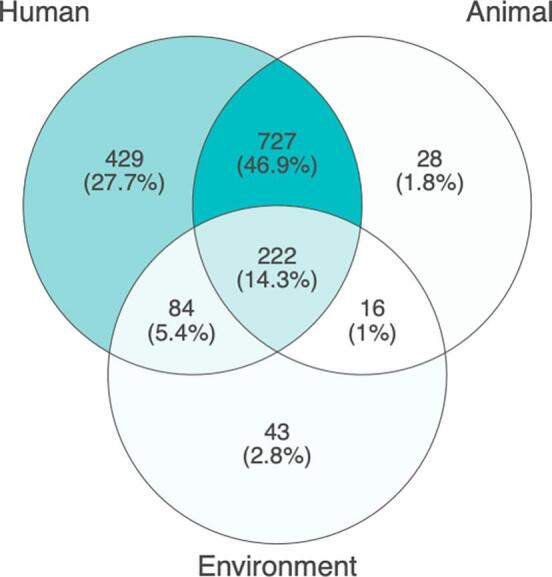
Distribution of One Health dimensions in problem definitions.

When examining only responses that identify one or more OH dimensions (human, animal, and environment; *n* = 1549), we find that 14.3% (*n* = 222) of respondents used a full OH problem definition in describing the issue of zoonotic spillovers (Fig. 2). To qualitatively interpret this result, it helps to examine the use of the other OH dimensions. For instance, the dominant use of human-animal (46.9%, *n* = 727) and human only dimensions (27.7%, *n* = 429) dwarf the use of a full OH problem definition. These results likely reflect OH's intellectual historical grounding in veterinary–medical collaborations in tandem with the people's looming concern for human health. Also of note, the paucity of use of animal and environment dimensions, with 5.4% (*n* = 84) identifying the human-environment connection, 2.8% (*n* = 43) citing the environment, 1.8% (*n* = 28) recognizing the role of animals, and 1% (*n* = 16) naming the link between animal-environment. Thus, the public, on the whole, has a strong human-centric perspective of zoonotic spillovers, and the environment is largely out of the general consciousness of people's thinking. This is significant because primary prevention strategies—such as habitat conservation, biodiversity protection, and ecological restoration—are most directly linked to environmental problem definitions. The relative absence of these perspectives indicates that the public is far less likely to prioritize prevention strategies rooted in ecological health.

**Fig. 3:**
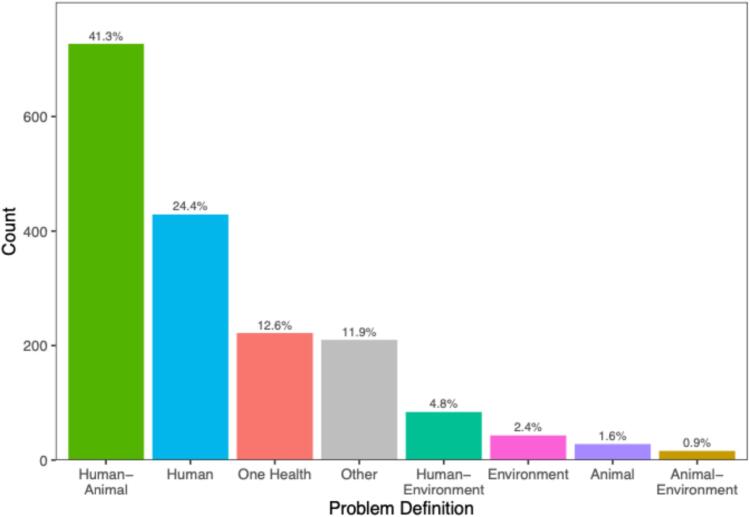
Distribution of non-, partial, and full One Health problem definitions.

Interestingly, not all respondents identified one of the three OH dimensions. There were also non-OH ideas posited by respondents (11.9%, *n* = 210; Fig. 3), for example: conspiracy theories, denial of spillovers occurring, natural selection, mutating viruses, and God's will. Indeed, when comparing “Other” non-OH problem definitions with those that included a OH dimension, about as many recognized a full OH problem definition as those who had no mention of OH dimensions. This is a strikingly high proportion given the prominence of OH discourse in policy and the recent establishment of a OH division within the Australian CDC. This may be indicative of a disconnect between the public's understanding and how governments view the problem.

In sum, these descriptive results reveal that a full OH problem definition is not widely prevalent in the public's mind. Instead, partial definitions dominate, which may constrain the range of solutions the public considers.

### 4.2 Does narrative content (i.e., heroes and solutions) vary when comparing a complete OH problem definition to other partial OH problem definitions?

To assess how narratives interact with problem definitions, we contrast the presence of heroes and solutions between OH and partial OH problem definitions. We begin by examining the results for the contrasts of OH problem definition to the partial OH problem definitions by heroes and then proceed with reporting results for solutions.

#### 4.2.1 Problem definition contrasts by heroes

**Fig. 4:**
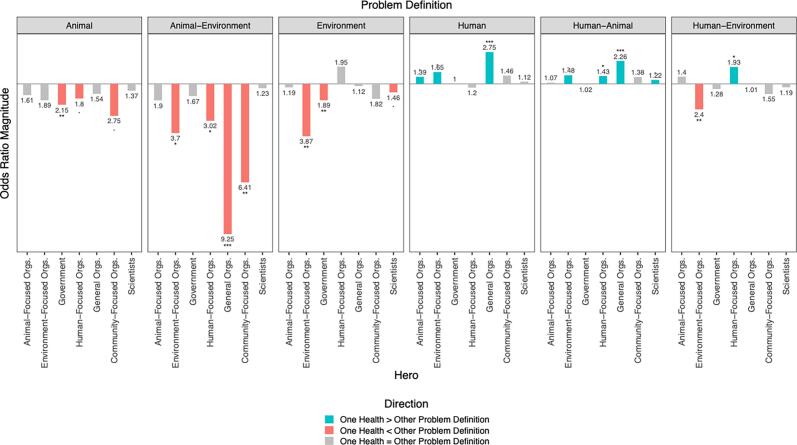
Estimated odds ratios for use of heroes when contrasting One Health with partial problem definitions. The relationship between rate of people choosing specific heroes depends on their problem definition. Numbers indicate the magnitude of multiplicative change in the odds of choosing a hero between two contrasting problem definitions. Stars indicate statistical significance of the difference in odds (*** = *p* < 0.001, ** = *p* < 0.01, * *p* < 0.05, • = *p* < 0.1). Colour and direction of the bars indicate which problem definition is the numerator of the odds ratio, with upwards bars comparing the odds of One Health to the other problem definitions (blue if significant) and downwards bars comparing the odds of other problem definitions to One Health (red if significant). Gray bars indicate there was statistically no significance difference in the odds of choosing a hero between the two problem definitions. Blue: The odds of One Health choosing this hero are __ times the odds of (_Problem Definition) choosing this hero. Red: The odds of (__Problem Definition) choosing this hero are __ times the odds of One Health choosing this hero. Gray: The odds of One Health choosing this hero are the same as (__Problem Definition). (For interpretation of the references to colour in this figure legend, the reader is referred to the web version of this article.)

Fig. 4 shows odds ratios for the presence of each hero category (Supplement A), comparing OH against partial problem definitions. Several patterns emerge:

At a glance, it is apparent that there are a number of positive, statistically significant odds ratios (blue bars) comparing OH to the three problem definitions that contain a ‘human’ dimension: Human, Human-Animal, Human-Environment. In other words, OH narratives are more likely to indicate certain hero types than these partial problem definitions. On the other hand, there are several negative, statistically significant odds ratios (red bars) comparing OH to problem definitions that contain no human dimension: Animal, Animal-Environment, and Environment. This represents OH narratives being less likely to indicate certain hero types than these partial problem definitions Overall, this can be taken as OH problem definition is narrated by different heroes compared to other problem definitions. What heroes are more likely to appear with which problem definition?

• The OH problem definition has higher odds of portraying heroes as Organizations across all OH dimensions (Animal-, Environment-, and Human-focused organizations), as well as organizations more generally, in contrast to the three partial problem definitions that contain the Human dimension (Human, Human-Animal, Human-Environment).
• Problem definitions that include the Environment (Animal-Environment, Human-Environment, and Environment) have higher odds of casting Environmentally focused organizations as the hero in contrast to a full OH problem definition.
• Problem definitions that include Animal or Environment (Animal, Animal-Environment) have higher odds of using Human- and Community-focused organizations contrast with a full OH problem definition.
• The single dimension problem definitions that include animal or environment (Animal, Environment) have higher odds of identifying Governmental heroes, in contrast to a full OH problem definition.
• OH problem definition has higher odds of selecting Scientists as heroes as compared to Human-Animal problem definition, but less likely to select them than to Environment problem definition.

In comparison with partial problem definitions, a OH problem definition was associated with a broader array of animal-, human-, and environment-oriented heroes, while being less likely to use government or community-focused actors. This suggests that respondents adopting a OH problem definition conceptualized zoonotic spillovers in a more comprehensive, systems way, recognizing a wider range of potential heroes compared to those using partial problem definitions.

#### 4.2.2 Problem definitions and solutions

**Fig. 5:**
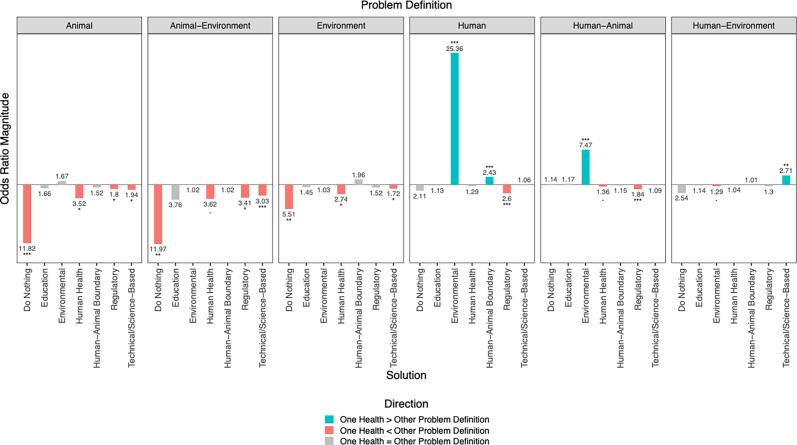
Estimated odds ratios of use of solutions when contrasting One Health with partial problem definitions. The relationship between rate of people choosing specific solutions depends on their problem definition (or vice versa). Numbers indicate the magnitude of multiplicative change in the odds of choosing a solution between two contrasting problem definitions. Stars indicate statistical significance of the difference in odds (*** = p < 0.001, ** = p < 0.01, * p < 0.05, • = p < 0.1). Colour and direction of the bars indicate which problem definition is the numerator of the odds ratio, with upwards bars comparing the odds of One Health to the other problem definitions (blue if significant) and downwards bars comparing the odds of other problem definitions to One Health (red if significant). Gray bars indicate there was statistically no significance difference in the odds of choosing a solution between the two problem definitions. Blue: The odds of One Health choosing this solution are __ times the odds of (__Problem Definition) choosing this solution. Red: The odds of (__Problem Definition) choosing this solution are __ times the odds of One Health choosing this solution. Gray: The odds of One Health choosing this solution are the same as (__Problem Definition). (For interpretation of the references to colour in this figure legend, the reader is referred to the web version of this article.)

Fig. 5 shows odds ratios for the presence of each solution category (Supplement A), comparing OH against partial problem definitions. Similar to the results of use of hero characters, solutions varied systematically with problem definitions. What solutions are more likely to appear with which problem definition?

• Environmental solutions had a higher likelihood of appearing in a OH problem definition in contrast to Human and Human-Animal definitions. This is logical, given the absence of the environmental dimension from Human and Human-Animal definitions and its presence in a full OH definition. However, Environmental solutions had a *lower* likelihood of appearing in OH definitions in contrast to Human–Environment definition, revealing the concentration of environmental solutions when focused on just two OH dimensions. Interestingly, there were equal odds (non-significance) of environmental solutions when contrasting OH problem definition to other Animal, Animal-Environment, and Environment alone definitions.
• OH problem definitions were less likely than other definitions to include regulatory solutions, which are typically aimed at preparation or mitigation centered solutions.
• There were equal odds of regulatory solutions when contrasting a OH problem definition to problem definitions that included the environment (Environment and Human-Environment).
• A OH problem definition also had a lower likelihood of including human health interventions, in contrast to other partial problem definitions focused on animals and the environment (Animal, Animal-Environment, Environment, Human-Animal). This is a surprise, given the absence of the human dimension in these problem definitions, compared with a OH problem definition. There were equal odds of human health solutions when contrasting OH problem definition with two problem definitions that include humans (Human, Human-Environment).
• OH problem definition also had a lower likelihood of including “doing nothing” as a solution compared with Animal, Environment, and Animal-Environment definitions. Likely this reflects the idea that doing nothing means less damage to animals and the environment. The equal odds of this solution for OH problem definition and the three partial definitions that contain the human dimension indicates the shared interest in doing something for human health in the face of zoonotic spillovers.
• Finally, Education appeared consistently across groups, with no significant differences. In other words, this is a shared solution across problem definitions.

Narrative content does vary somewhat by problem definition when comparing use of heroes and solutions in OH problem definitions in contrast to their use in partial problem definitions.

### 4.3 Is the narrative content congruent with the problem definitions?

**Fig. 6:**
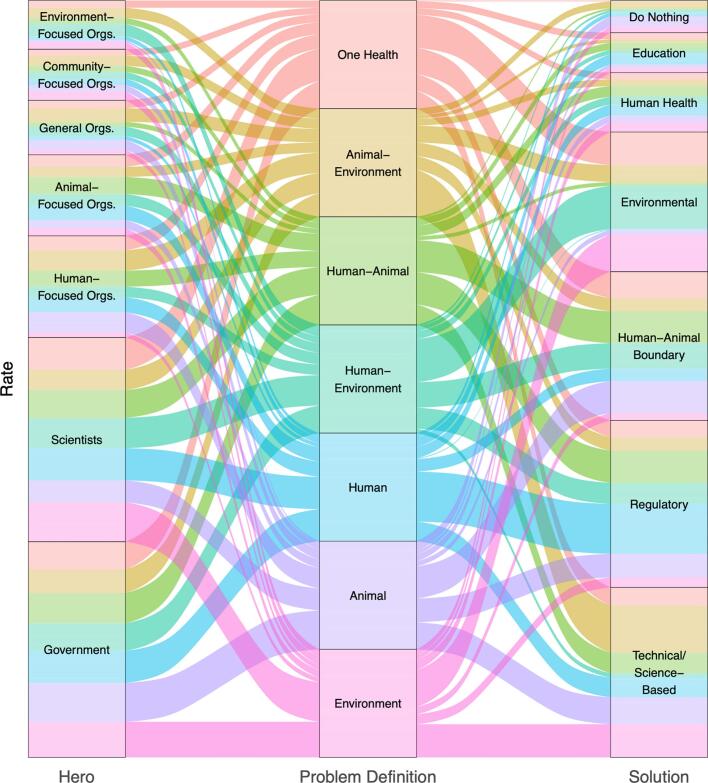
Alluvial plot of hero and solution use scaled by problem definition group size. Flows are colored by Problem Definition and scaled by group size or rate (versus count). RED – One Health; BROWN – Animal-Environment; LIGHT GREEN – Human-Animal; BLUE GREEN – Human-Environment; BLUE – Human; PURPLE - Animal; PINK-Environment. (For interpretation of the references to colour in this figure legend, the reader is referred to the web version of this article.)

Theoretically, problem definitions should align with the solutions that respondents attach to them [8], [28]. What is less known is the role of the hero in actualizing the solutions. To explore these relationships, we look descriptively at the assignment of heroes and solutions by problem definition. The alluvial plot (Fig. 6) below reveals the flow between problem definitions, heroes, and solutions, proportional to the size of the problem definition group to allow for comparison of magnitude.

Examining the proportional assignment of solutions by problem definition, a distinct pattern emerges that supports the concept that problem definition is congruent with solutions. Environmental solutions (e.g., address land use change, protect forests, address climate change) are the primary moral of the story for problem definitions that include the environment: One Health, Human-Environment, and Environment. Notably missing is Animal-Environment problem definition, that aligns with Animal and Environment problem definitions to largely posit Technical/science-based solutions (e.g., vaccines, surveillance, research). The human-centered partial problem definitions (Human, Human-Environment, and Human-Animal) shared a strong narration of Regulatory solutions (e.g., border control, biosecurity, food safety standards). This reveals the need for rules to promote healthy behaviors. Finally, the solution focused on Human-animal boundaries (e.g., quarantine, reduce meat consumption, address human-animal contact) is dominant in One Health, Human-Animal, Human-Environment, and Animal problem definitions, revealing a shared solution across even the siloed dimensions of One Health. In all, the primary solutions coming from how people define the problem of zoonotic spillovers is congruent with the problem definitions.

The frequency of different heroes associated with these problem definitions reveals an interesting story. The singular dimensions of problem definitions (Human, Animal, and Environment) in tandem with the addition of the human dimension (Human-Animal and Human-Environment), all converge on scientists and government as the fixers to the problem. And yet, while the same heroes are narrated to fix the problem of spillovers, *how* they are to fix the problem (the solution) varies by problem definition. Additionally, both the OH and the Animal-Environment problem definitions do not have a dominant hero, but, rather, a somewhat equal distribution across multiple heroes. This is likely reflective of a more systems way of thinking–that the solutions must include multisector collaboration.

We observe congruence between problem definitions and solutions; yet, the use of heroes reveals two important dynamics. First, most partial problem definitions identify government and scientists as the actors responsible for fixing the problem, yet they assign these characters different solution pathways depending on how the problem is defined. This may explain tensions between the public's desire for government and scientific action and the divergent approaches implied by competing problem definitions. Second, the relatively equally shared character types serving as hero for OH and Animal-Environment problem definitions organically reveal the multisectoral, multidisciplinary, systems approach that is the bedrock for the OH approach. Together, these dynamics underscore how narrative constructions reveal the complex nature of OH governance, with competing problem definitions leading to different solution sets and heroes illuminating both the tensions and the integrative potential inherent in a OH approach.

## 5 Conclusion

As governments move from OH strategic plans toward concrete policy and implementation, understanding public perceptions of OH is critical. Our study demonstrates that public uptake of fully integrated OH understanding of zoonotic spillovers remains limited; only a small fraction of respondents articulated definitions that simultaneously included humans, animals, and the environment. Instead, partial problem definitions dominate, and these often lead to solutions that are reactive—such as technical containment—rather than preventive measures like ecological protection. Narratives also varied systematically by problem definition. OH narratives emphasized diverse heroes and environmental solutions, while human-centered definitions tended to privilege regulatory interventions. Evidence of narrative congruence was apparent, as problem definitions tended to align logically with their corresponding solutions. Interestingly, the use of the same dominant heroes (government and scientists) for most partial problem definitions may reveal the root of some public dissatisfaction, as the accompanying solutions are divergent, depending on the problem definition. Additionally, the broad array of heroes for a OH problem definition is congruent with the Quadripartite's assertion that a OH system needs multisectoral and multidisciplinary input to solve OH issues [1]. Taken together, these findings suggest that public narratives about zoonotic spillovers remain fragmented, with limited adoption of the integrative OH perspective. Such fragmentation risks undermining prevention-oriented policy, as siloed narratives may generate inconsistent or reactive solutions. Strengthening public understanding of OH and fostering integrative narratives could therefore play an essential role in building political legitimacy and public support for multisectoral policies. This is achieved through a consistent, systems-oriented communication strategy as government officials, scientists, and others discuss and describe OH issues to the general public. Ultimately, the development of clear, coherent narratives that connect integrated OH problem definitions with appropriate solutions will be crucial for securing broad-based support and ensuring effective policy to prevent future pandemics.

## CRediT authorship contribution statement

**Elizabeth A. Shanahan:** Writing – original draft, Validation, Supervision, Project administration, Funding acquisition, Formal analysis, Conceptualization. **Xin Han:** Writing – review & editing, Validation, Formal analysis, Conceptualization. **Sarah Salam:** Writing – original draft, Formal analysis. **Savanna Washburn:** Writing – original draft, Validation, Investigation, Formal analysis. **Sally K. Slipher:** Writing – original draft, Visualization, Methodology, Formal analysis, Data curation.

## Declaration of competing interest

The authors declare the following financial interests/personal relationships which may be considered as potential competing interests:

Elizabeth A. Shanahan reports financial support was provided by 10.13039/100000001National Science Foundation (Award number: 2231624). Sally Slipher reports that financial support was provided by Social Data Analysis & Collection Services (RRID:SCR_026326) and the National Institute of General Medical Sciences of the National Institutes of Health under Award Number P20GM103474 and 10.13039/100009501Montana State University. Other authors declare that they have no known competing financial interests or personal relationships that could have appeared to influence the work reported in this paper.

## Data Availability

Data will be made available on request.
