# Supplementary material for: One Health in the public mind: Narrative constructions of zoonotic spillovers
Source: One Health. 2026 Apr 14;22:101411. doi: 10.1016/j.onehlt.2026.101411 (PMC13133971; doi:10.1016/j.onehlt.2026.101411)
Supplement: Supplementary file 1 — Supplementary material [file mmc1.docx]

**SUPPLEMENT A**

**CODEBOOKS FOR PROBLEM DEFINITIONS, HEROES, AND SOLUTIONS**

**OH PROBLEM DEFINITIONS**

| ONE HEALTH |
| --- |
| - Human--Animal/wild--Environment/biotic |
| - Human--Animal/wild--Environment/abiotic |
| - Human--Animal/wild--Environment/both |
| - Human--Animal/wild--Environment/unspecified |
| - Human--Animal/domestic--Environment/biotic |
| - Human--Animal/domestic--Environment/abiotic |
| - Human--Animal/domestic--Environment/both |
| - Human--Animal/domestic--Environment/unspecified |
| - Human--Animal/both--Environment/biotic |
| - Human--Animal/both--Environment/abiotic |
| - Human--Animal/both--Environment/both |
| - Human--Animal/both--Environment/unspecified |
| - Human--Animal/unspecified--Environment/biotic |
| - Human--Animal/unspecified--Environment/abiotic |
| - Human--Animal/unspecified--Environment/both |
| - Human--Animal/unspecified--Environment/unspecified |

| SINGLE DIMENSION |
| --- |
| - Human |
| - Animal/wild |
| - Animal/domestic |
| - Animal/both |
| - Animal/unspecified |
| - Environment/biotic |
| - Environment/abiotic |
| - Environment/both |
| - Environment/unspecified |

| TWO ONE HEALTH DIMENSIONS |
| --- |
| - Human--Animal/wild |
| - Human--Animal/domestic |
| - Human--Animal/both |
| - Human--Animal/unspecified |
| - Human--Environment/biotic |
| - Human--Environment/abiotic |
| - Human--Environment/both |
| - Human--Environment/unspecified |
| - Animal/wild--Environment/biotic |
| - Animal/wild--Environment/abiotic |
| - Animal/wild--Environment/both |
| - Animal/wild--Environment/unspecified |
| - Animal/domestic--Environment/biotic |
| - Animal/domestic--Environment/abiotic |
| - Animal/domestic--Environment/both |
| - Animal/domestic--Environment/unspecified |
| - Animal/both--Environment/biotic |
| - Animal/both--Environment/abiotic |
| - Animal/both--Environment/both |
| - Animal/both--Environment/unspecified |
| - Animal/unspecified--Environment/biotic |
| - Animal/unspecified--Environment/abiotic |
| - Animal/unspecified--Environment/both |
| - Animal/unspecified--Environment/unspecified |

| OTHER |
| --- |
| - Conspiracy/Not Happening |
| - How it is/Inevitable |
| - Viral mutation/More viruses/Natural selection |
| - Increased detection or awareness/Always happened |
| - God/Spiritual |

| EXCLUDED FROM ANALYSIS |
| --- |
| - Do not know |
| - Cannot determine |
| - Nonsensical response |

**HEROES**

| GOVERNMENT   - Governing System_global independent unit - Governing System_confederation - Government_general - Government_federal - Government_state - Government_local - Government_agriculture/domestic - Government_wildlife - Government_human health - Government_environment |
| --- |
| COMMUNITY-FOCUSED ORGS   - People (local communities, everyone, self)   HUMAN FOCUSED ORGS   - Orgs_medical/health - Professionals_health workers - Professionals_educators - Professionals_private business/individuals   ENVIRONMENT FOCUSED ORGS   - Orgs_environment - Professionals_conservationists/environmentalist   ANIMAL FOCUSED ORGS   - Orgs_wildlife - Orgs_agriculture/domestic animals - Professionals_veterinarians - Professionals_wildlife/zoos - Professionals_animal management/husbandry/farmers   ORGS (GENERAL)   - Orgs _general - Orgs_WHO |
| SCIENTISTS   - Scientists_environment - Scientists_wildlife - Scientists_animals (domestic) - Scientists_medical/human health - Scientists_general - Scientists_government (e.g., CSIRO, CDC) - Scientists_specific field |
| OTHER   - God |
| - Habitat |
| - Information |

**SOLUTIONS**

HUMAN-ANIMAL BOUNDARY

- ban_wet_markets
- cull_animals
- control_population_wildlife
- no_animal_lab_testing
- quarantine
- reduce_human_animal_contact
- stop_reduce_eating_keeping_feeding_exotic_wild_animals
- animal_border_in_migration_issues_incl_import_export_food
- stop_reduce_eating_domestic_animals
- stop_reduce_eating_meat_general

TECHNICAL/SCIENCE-BASED

- vaccines_unspecified
- vaccines_people
- vaccines_animals
- medication
- surveillance_testing_unspecified
- surveillance_tesing_animals
- surveillance_testing_people
- research_fund_conduct_develop
- leave_it_to_the_experts

REGULATORY

- border_control_general
- control_population_human
- control_population_unspecified
- human_travel_protocols_disinfect_luggage_and_restrictions
- regulate_close_fine_China_Asia_India_Russia
- regulate_lab_testing_to_reduce_spillovers
- regulations_laws_govt_biosecurity_control
- standards_regulations_for_handling_live_animals_transport_pets_farmers_wildlife_carers
- food_safety_regulations_incl_processing_and_handling
- protect_animals

ENVIRONMENTAL

- reduce_be_careful_about_land_use_change
- increase_protect_forests_wildlife_habitat
- address_climate_change

HUMAN HEALTH

- good_hygiene
- better_human_health_living_conditions
- more_organic_farming_practices
- PPE
- reduce_use_of_antibiotics
- eat_shop_local
- support_listen_to_farmers

EDUCATION

- communication_during_crisis
- education

DO NOTHING

- Dont_interfere_herd_immunity_leave_alone
- nothing_you_can_do_to_stop_it

**SUPPLEMENT B**

**INTERCODER RELIABILITY**

| **SOLUTIONS** | | | | | | | | |
| --- | --- | --- | --- | --- | --- | --- | --- | --- |
|  | % Agree | Scott's Pi | Cohen's Kappa | Krippendorff's Alpha (nominal) | N  Agree | N  Disagree | N  Cases | N  Decisions |
| Cull_animals | 100% | 1 | 1 | 1 | 99 | 0 | 99 | 198 |
| vaccines_people | 100% | undefined* | undefined* | undefined* | 99 | 0 | 99 | 198 |
| vaccines_animals | 100% | undefined* | undefined* | undefined* | 99 | 0 | 99 | 198 |
| vaccines_unspecified | 100% | 1 | 1 | 1 | 99 | 0 | 99 | 198 |
| surveillance/testing_people | 100% | undefined* | undefined* | undefined* | 99 | 0 | 99 | 198 |
| surveillance/testing_animals | 100% | 1 | 1 | 1 | 99 | 0 | 99 | 198 |
| surveillance/testing_unspecified | 97% | -0.015 | 0 | -0.01 | 96 | 3 | 99 | 198 |
| ban wet markets | 100% | 1 | 1 | 1 | 99 | 0 | 99 | 198 |
| standards/regulations for handling live animals (transport, pets, farmers, wildlife carers) | 97% | 0.753 | 0.754 | 0.754 | 96 | 3 | 99 | 198 |
| stop/reduce eating domestic animals | 100% | undefined* | undefined* | undefined* | 99 | 0 | 99 | 198 |
| stop/reduce eating-keeping-feeding exotic/wild animals | 99% | 0.904 | 0.904 | 0.904 | 98 | 1 | 99 | 198 |
| stop/reduce eating meat_general | 100% | undefined* | undefined* | undefined* | 99 | 0 | 99 | 198 |
| food safety/ regulations (incl processing and handling) | 99% | 0.795 | 0.795 | 0.796 | 98 | 1 | 99 | 198 |
| regulations-laws-gov't-biosecurity ('control') | 98% | 0.789 | 0.79 | 0.79 | 97 | 2 | 99 | 198 |
| no animal lab testing | 100% | 1 | 1 | 1 | 99 | 0 | 99 | 198 |
| regulate/close/fine China/Asia/India/ Russia | 100% | 1 | 1 | 1 | 99 | 0 | 99 | 198 |
| reduce human-animal contact | 98% | 0.889 | 0.889 | 0.889 | 97 | 2 | 99 | 198 |
| reduce/be careful about land use change | 99% | 0.884 | 0.884 | 0.884 | 98 | 1 | 99 | 198 |
| increase/protect forests, wildlife habitat | 97% | 0.556 | 0.556 | 0.558 | 96 | 3 | 99 | 198 |
| good hygiene | 99% | 0.904 | 0.904 | 0.904 | 98 | 1 | 99 | 198 |
| better human health/living conditions | 99% | -0.005 | 0 | 0 | 98 | 1 | 99 | 198 |
| PPE | 100% | undefined* | undefined* | undefined* | 99 | 0 | 99 | 198 |
| research--fund, conduct, develop | 98% | 0.878 | 0.878 | 0.878 | 97 | 2 | 99 | 198 |
| education | 98% | 0.789 | 0.79 | 0.79 | 97 | 2 | 99 | 198 |
| communication (during crisis) | 100% | undefined* | undefined* | undefined* | 99 | 0 | 99 | 198 |
| human travel protocols (disinfect luggage) and restrictions | 100% | 1 | 1 | 1 | 99 | 0 | 99 | 198 |
| animal border/in-migration issues (incl import/export & food) | 99% | -0.005 | 0 | 0 | 98 | 1 | 99 | 198 |
| border control_general | 100% | undefined* | undefined* | undefined* | 99 | 0 | 99 | 198 |

| **HEROES** | | | | | | | | |
| --- | --- | --- | --- | --- | --- | --- | --- | --- |
|  | % Agree | Scott's Pi | Cohen's Kappa | Krippendorff's Alpha (nominal) | N  Agree | N  Disagree | N  Cases | N  Decisions |
| Governing System_global independent unit | 99 | -0.00337 | 0 | 0 | 148 | 1 | 149 | 298 |
| Governing System_confederation | 98 | 0.71682 | 0.716909 | 0.71777 | 146 | 3 | 149 | 298 |
| Government_general | 95 | 0.85571 | 0.855959 | 0.856194 | 142 | 7 | 149 | 298 |
| Government_federal | 97 | 0.763492 | 0.764241 | 0.764286 | 145 | 4 | 149 | 298 |
| Government_state | 100 | 1 | 1 | 1 | 149 | 0 | 149 | 298 |
| Government_local | 100 | 1 | 1 | 1 | 149 | 0 | 149 | 298 |
| Government_agriculture/domestic animals | 100 | 1 | 1 | 1 | 149 | 0 | 149 | 298 |
| Government_human health | 99 | 0.885429 | 0.885473 | 0.885813 | 148 | 1 | 149 | 298 |
| Government_wildlife | 99 | 0.493197 | 0.493197 | 0.494898 | 147 | 2 | 149 | 298 |
| Government_environment | 99 | -0.00337 | 0 | 0 | 148 | 1 | 149 | 298 |
| Orgs _general | 99 | 0.663277 | 0.663657 | 0.664407 | 148 | 1 | 149 | 298 |
| Orgs _medical/health | 100 | 1 | 1 | 1 | 149 | 0 | 149 | 298 |
| Orgs_wildlife | 99 | 0.885429 | 0.885473 | 0.885813 | 148 | 1 | 149 | 298 |
| Orgs_environment | 99 | 0.663277 | 0.663657 | 0.664407 | 148 | 1 | 149 | 298 |
| Orgs _agriculture/domestic animals | 100 | 1 | 1 | 1 | 149 | 0 | 149 | 298 |
| Orgs_WHO | 99 | 0.853706 | 0.853778 | 0.854197 | 148 | 1 | 149 | 298 |
| Scientists_general | 98 | 0.938161 | 0.938166 | 0.938369 | 146 | 3 | 149 | 298 |
| Scientists_government (e.g., CSIRO, CDC) | 100 | 1 | 1 | 1 | 149 | 0 | 149 | 298 |
| Scientists_environment | 100 | undefined* | undefined* | undefined* | 149 | 0 | 149 | 298 |
| Scientists_wildlife | 99 | 0.9298 | 0.929816 | 0.930035 | 148 | 1 | 149 | 298 |
| Scientists_animals (domestic) | 100 | undefined* | undefined* | undefined* | 149 | 0 | 149 | 298 |
| Scientists_medical/human health | 100 | 1 | 1 | 1 | 149 | 0 | 149 | 298 |
| Scientists_specific field | 97 | 0.427143 | 0.429119 | 0.429066 | 144 | 5 | 149 | 298 |
| Professionals_health workers | 100 | 1 | 1 | 1 | 149 | 0 | 149 | 298 |
| Professionals_veterinarians | 100 | 1 | 1 | 1 | 149 | 0 | 149 | 298 |
| Professionals_animal management/husbandry/farmers | 100 | 1 | 1 | 1 | 149 | 0 | 149 | 298 |
| Professionals_wildlife/zoos | 97 | 0.735816 | 0.735816 | 0.736702 | 145 | 4 | 149 | 298 |
| Professionals_conservationists/environmentalist | 99 | 0.853706 | 0.853778 | 0.854197 | 148 | 1 | 149 | 298 |
| Professionals_educators | 99 | 0.493197 | 0.493197 | 0.494898 | 147 | 2 | 149 | 298 |
| Professionals_private business/individuals | 100 | undefined* | undefined* | undefined* | 149 | 0 | 149 | 298 |
| People (local communities, everyone, myself) | 95 | 0.526984 | 0.527359 | 0.528571 | 141 | 8 | 149 | 298 |
| God | 100 | 1 | 1 | 1 | 149 | 0 | 149 | 298 |
| Habitat | 99 | 0.493197 | 0.494915 | 0.494898 | 147 | 2 | 149 | 298 |
| Information | 99 | -0.00337 | 0 | 0 | 148 | 1 | 149 | 298 |
| Don’t know | 99 | 0.942248 | 0.942248 | 0.942442 | 147 | 2 | 149 | 298 |
| No one/as it is not a problem | 98 | 0.389761 | 0.390177 | 0.391809 | 146 | 3 | 149 | 298 |
| No hero presented | 93 | 0.339539 | 0.344767 | 0.341755 | 139 | 10 | 149 | 298 |
| Cannot determine | 95 | 0.198001 | 0.198309 | 0.200692 | 142 | 7 | 149 | 298 |
| Nonsensical response | 99 | 0.663277 | 0.663657 | 0.664407 | 148 | 1 | 149 | 298 |

**SUPPLEMENT C.**

**EXAMPLES OF OBSERVATIONS AND UNIQUE OBSERVATIONS BY SUBJECT**

| **UID** | **Hero** | **Problem** | **Solution** |
| --- | --- | --- | --- |
| 52 | Government | Human | Regulatory |
| 53 | Government | Human | Regulatory |
| 53 | Human- Focused Orgs. | Human | Regulatory |
| 53 | Scientists | Human | Regulatory |
| 53 | Animal- Focused Orgs. | Human | Regulatory |
| 291 | Government | Human-Animal | Human-Animal Boundary |
| 291 | Government | Human-Animal | Human Health |
| 318 | Government | One Health | Human-Animal Boundary |
| 318 | Government | One Health | Human Health |
| 318 | Government | One Health | Education |
| ... | ... | ... | ... |

**Observations by subject: n = 3551 observations; n = 1549 subjects**

**Unique combinations: n = 255 unique combinations of categories**

| **Hero** | **Problem** | **Solution** | **Rate** | **Count** | **GroupSize** |
| --- | --- | --- | --- | --- | --- |
| Environment-Focused Orgs. | Human-Animal | Do Nothing | 0.0005763689 | 1 | 1,735 |
| Animal- Focused Orgs. | Human | Environmental | 0.0012610340 | 1 | 793 |
| Community- Focused Orgs. | Human | Environmental | 0.0012610340 | 1 | 793 |
| Environment- Focused Orgs. | One Health | Human Health | 0.0015552100 | 1 | 643 |
| Community- Focused Orgs. | One Health | Do Nothing | 0.0015552100 | 1 | 643 |
| Community- Focused Orgs. | One Health | Education | 0.0015552100 | 1 | 643 |
| Scientists | One Health | Do Nothing | 0.0015552100 | 1 | 643 |
| Human- Focused Orgs. | Human-Animal | Do Nothing | 0.0017291066 | 3 | 1,735 |
| General Orgs. | Human-Animal | Do Nothing | 0.0017291066 | 3 | 1,735 |
| Community- Focused Orgs. | Human-Animal | Environmental | 0.0017291066 | 3 | 1,735 |
| ... | ... | ... | ... | ... | ... |

**SUPPLEMENT D**

**DEMOGRAPHICS BY PROBLEM DEFINITION**

| Problem Definition | Overall   N = 1,549 | One Health   N = 222 | Animal-Environment   N = 16 | Human-Animal   N = 727 | Human-Environment   N = 84 | Human   N = 429 | Animal   N = 28 | Environment   N = 43 |
| --- | --- | --- | --- | --- | --- | --- | --- | --- |
| State |  |  |  |  |  |  |  |  |
| NSW | 579 (37%) | 83 (37%) | 6 (38%) | 287 (39%) | 28 (33%) | 151 (35%) | 7 (25%) | 17 (40%) |
| Victoria | 513 (33%) | 68 (31%) | 6 (38%) | 245 (34%) | 29 (35%) | 143 (33%) | 11 (39%) | 11 (26%) |
| QLD | 457 (30%) | 71 (32%) | 4 (25%) | 195 (27%) | 27 (32%) | 135 (31%) | 10 (36%) | 15 (35%) |
| Urbanization |  |  |  |  |  |  |  |  |
| Metropolitan | 914 (59%) | 137 (62%) | 12 (75%) | 427 (59%) | 49 (58%) | 250 (58%) | 13 (46%) | 26 (60%) |
| Regional Center | 416 (27%) | 47 (21%) | 3 (19%) | 208 (29%) | 23 (27%) | 115 (27%) | 11 (39%) | 9 (21%) |
| Rural | 198 (13%) | 34 (15%) | 1 (6.3%) | 83 (11%) | 12 (14%) | 57 (13%) | 4 (14%) | 7 (16%) |
| Remote | 21 (1.4%) | 4 (1.8%) | 0 (0%) | 9 (1.2%) | 0 (0%) | 7 (1.6%) | 0 (0%) | 1 (2.3%) |
| Age | 54 (18) | 53 (17) | 49 (12) | 54 (19) | 51 (15) | 56 (18) | 49 (17) | 54 (17) |
| Gender |  |  |  |  |  |  |  |  |
| Male | 708 (46%) | 87 (39%) | 8 (50%) | 331 (46%) | 38 (45%) | 209 (49%) | 18 (64%) | 17 (40%) |
| Female | 841 (54%) | 135 (61%) | 8 (50%) | 396 (54%) | 46 (55%) | 220 (51%) | 10 (36%) | 26 (60%) |
| Other | 0 (0%) | 0 (0%) | 0 (0%) | 0 (0%) | 0 (0%) | 0 (0%) | 0 (0%) | 0 (0%) |
| Prefer Not To Say | 0 (0%) | 0 (0%) | 0 (0%) | 0 (0%) | 0 (0%) | 0 (0%) | 0 (0%) | 0 (0%) |
| Education |  |  |  |  |  |  |  |  |
| Primary | 5 (0.3%) | 0 (0%) | 0 (0%) | 2 (0.3%) | 0 (0%) | 3 (0.7%) | 0 (0%) | 0 (0%) |
| Year 7 to Year 9 | 42 (2.7%) | 2 (0.9%) | 0 (0%) | 19 (2.6%) | 3 (3.6%) | 18 (4.2%) | 0 (0%) | 0 (0%) |
| Year 10 | 117 (7.6%) | 8 (3.6%) | 2 (13%) | 62 (8.5%) | 6 (7.1%) | 32 (7.5%) | 2 (7.1%) | 5 (12%) |
| Year 11 | 43 (2.8%) | 3 (1.4%) | 1 (6.3%) | 20 (2.8%) | 1 (1.2%) | 17 (4.0%) | 1 (3.6%) | 0 (0%) |
| Year 12 | 219 (14%) | 39 (18%) | 2 (13%) | 101 (14%) | 8 (9.5%) | 60 (14%) | 5 (18%) | 4 (9.3%) |
| Non-Trade | 117 (7.6%) | 20 (9.0%) | 0 (0%) | 48 (6.6%) | 6 (7.1%) | 41 (9.6%) | 2 (7.1%) | 0 (0%) |
| Trade | 183 (12%) | 19 (8.6%) | 1 (6.3%) | 82 (11%) | 9 (11%) | 66 (15%) | 4 (14%) | 2 (4.7%) |
| Associate | 195 (13%) | 25 (11%) | 0 (0%) | 96 (13%) | 5 (6.0%) | 53 (12%) | 5 (18%) | 11 (26%) |
| Undergrad | 55 (3.6%) | 4 (1.8%) | 0 (0%) | 28 (3.9%) | 3 (3.6%) | 19 (4.4%) | 1 (3.6%) | 0 (0%) |
| Bachelor | 392 (25%) | 76 (34%) | 8 (50%) | 185 (25%) | 25 (30%) | 83 (19%) | 3 (11%) | 12 (28%) |
| Postgrad | 181 (12%) | 26 (12%) | 2 (13%) | 84 (12%) | 18 (21%) | 37 (8.6%) | 5 (18%) | 9 (21%) |
| Income |  |  |  |  |  |  |  |  |
| Less than $25,000 | 136 (8.8%) | 18 (8.1%) | 1 (6.3%) | 67 (9.2%) | 4 (4.8%) | 36 (8.4%) | 3 (11%) | 7 (16%) |
| $25,000 to $39,999 | 241 (16%) | 27 (12%) | 1 (6.3%) | 121 (17%) | 7 (8.3%) | 81 (19%) | 1 (3.6%) | 3 (7.0%) |
| $40,000 to $54,999 | 195 (13%) | 24 (11%) | 1 (6.3%) | 89 (12%) | 14 (17%) | 59 (14%) | 5 (18%) | 3 (7.0%) |
| $55,000 to $69,999 | 172 (11%) | 29 (13%) | 2 (13%) | 76 (10%) | 11 (13%) | 44 (10%) | 4 (14%) | 6 (14%) |
| $70,000 to $84,999 | 145 (9.4%) | 22 (9.9%) | 3 (19%) | 72 (9.9%) | 7 (8.3%) | 33 (7.7%) | 4 (14%) | 4 (9.3%) |
| $85,000 to $99,999 | 134 (8.7%) | 26 (12%) | 3 (19%) | 61 (8.4%) | 4 (4.8%) | 35 (8.2%) | 3 (11%) | 2 (4.7%) |
| $100,000 to $114,999 | 77 (5.0%) | 8 (3.6%) | 0 (0%) | 40 (5.5%) | 3 (3.6%) | 20 (4.7%) | 3 (11%) | 3 (7.0%) |
| $115,000 to $129,999 | 63 (4.1%) | 13 (5.9%) | 0 (0%) | 31 (4.3%) | 5 (6.0%) | 13 (3.0%) | 0 (0%) | 1 (2.3%) |
| $130,000 to $149,999 | 112 (7.2%) | 15 (6.8%) | 1 (6.3%) | 53 (7.3%) | 7 (8.3%) | 27 (6.3%) | 2 (7.1%) | 7 (16%) |
| $150,000 to $174,999 | 46 (3.0%) | 3 (1.4%) | 0 (0%) | 26 (3.6%) | 0 (0%) | 15 (3.5%) | 1 (3.6%) | 1 (2.3%) |
| $175,000 to $199,999 | 51 (3.3%) | 11 (5.0%) | 2 (13%) | 19 (2.6%) | 5 (6.0%) | 11 (2.6%) | 0 (0%) | 3 (7.0%) |
| $200,000 or more | 73 (4.7%) | 13 (5.9%) | 2 (13%) | 29 (4.0%) | 8 (9.5%) | 20 (4.7%) | 1 (3.6%) | 0 (0%) |
| Don't Know | 18 (1.2%) | 0 (0%) | 0 (0%) | 6 (0.8%) | 0 (0%) | 11 (2.6%) | 1 (3.6%) | 0 (0%) |
| Prefer Not To Say | 86 (5.6%) | 13 (5.9%) | 0 (0%) | 37 (5.1%) | 9 (11%) | 24 (5.6%) | 0 (0%) | 3 (7.0%) |
| Have any Children (yes) | 354 (23%) | 46 (21%) | 3 (19%) | 173 (24%) | 25 (30%) | 88 (21%) | 8 (29%) | 11 (26%) |
| *Mean (SD) reported for continuous variables.* | | | | | | | | |
| *n (%) reported for categorical variables.* | | | | | | | | |

**SUPPLEMENT E**

**MODEL SUMMARY**

ANOVA of covariates/interactions from final model:

| **Analysis of Deviance Table (Type III tests)** | |
| --- | --- |
| **Term** | ***p* Value** |
| Hero | 0.035 |
| Problem Definition | < 0.001 |
| Solution | < 0.001 |
| Hero:Problem Definition | < 0.001 |
| Problem Definition:Solution | < 0.001 |
| Hero:Solution | 0.057 |

Measures of model fit from final model:

$$\log\left( \mathbb{E}\left[ Y \right] \right)=\beta_{0}+\beta_{1}\cdot\text{Hero}+\beta_{2}\cdot\text{Problem}+\beta_{3}\cdot\text{Solution}+\beta_{4}\cdot\left( \text{Hero}\times\text{Problem} \right)+\beta_{5}\cdot\left( \text{Hero}\times\text{Solution} \right)+\beta_{6}\cdot\left( \text{Problem}\times\text{Solution} \right)+log\left( \text{GroupSize} \right)$$

| **Metric** | **Value** |
| --- | --- |
| AIC | 1,233.965 |
| Residual Deviance | 66.847 |
| Null Deviance | 3,519.439 |
| Residual DF | 128.000 |
| Null DF | 254.000 |
| Log-Likelihood | -489.983 |
| McFadden's Pseudo-R² | 0.862 |
| Dispersion (Pearson χ² / DF) | 0.553 |
| Number of Observations | 255.000 |

Likelihood Ratio test comparing with 3-way interaction model:

| **Model** | **Residual DF** | **Residual Deviance** | **DF** | **Deviance** | **p Value** |
| --- | --- | --- | --- | --- | --- |
| Full (3-way interaction) | 0 | 0.00000000000005506706 |  |  |  |
| Reduced (2-way interactions only) | 128 | 66.84669461260675404901 | -128 | -66.84669 | 0.9999983 |

**SUPPLEMENT F**

**GROUP SIZES FOR OBSERVATIONS**

**Group sizes (for n = 3551 observations):**

| **Problem** | **n** |
| --- | --- |
| One Health | 643 |
| Animal | 64 |
| Animal-Environment | 32 |
| Environment | 88 |
| Human | 793 |
| Human-Animal | 1,735 |
| Human-Environment | 196 |
